# Receptor-interacting protein kinase 1 (RIPK1) regulates cervical cancer cells via NF-κB–TNF-α pathway: An in vitro study

**DOI:** 10.1016/j.tranon.2023.101748

**Published:** 2023-07-27

**Authors:** Wenqi Bai, Fengjie Cui, Zihan Wang, Xianhua Gu, Xiaojing Fang, Li Zhou, Suyang Guo

**Affiliations:** a Departments of Oncology Gynecology, The First Affiliated Hospital of Bengbu Medical College, No. 287 Changhuai Road, Bengbu 233004, China; b Department of Oncology Surgery, The First Affiliated Hospital of Bengbu Medical College, Bengbu, China

**Keywords:** Eceptor-interacting protein kinase 1, Cervical carcinoma, NF-κB, Apoptosis, Targeted therapy

## Abstract

**Introduction:**

Cervical cancer (CC) is associated with high morbidity and mortality rates in women. Members of the receptor-interacting protein kinase (RIPK) family are important regulators of inflammation and cell death. However, the characteristics, molecular functions, and expression mechanisms of RIPK1 in CC remain unclear.

**Material and methods:**

To determine whether RIPK1 can be used for targeted therapy of CC, we assessed the clinical importance, biological function, and potential impact of RIPK1 in CC in 50 patients with CC. We utilized immunohistochemical staining, transfection, western blotting, cell counting kit-8 assay, colony formation assay, and wound healing assays among others, to elucidate the role of RIPK1 in CC.

**Results:**

RIPK1 expression was higher in tumor tissues than in paracancerous tissues. Poor prognosis of CC was linked to RIPK1 upregulation. Furthermore, silencing RIPK1 significantly inhibited the proliferation, migration, and invasion of CC cells in vitro. We also established that RIPK1 increased cell migration, invasion, and multiplication by regulating nuclear factor kappa-B (NF-κB) and tumor necrosis factor (TNF).

**Discussion:**

RIPK1 activates NF-κB and regulates TNF release to enhance the proliferation and spread of CC cells while suppressing their apoptosis. Therefore, RIPK1 plays a key role in the formation and progression of CC and is a potential target for CC treatment.

## Highlights

• The role of RIPK1 in cervical cancer (CC) remains unclear.
• We studied the relevance, role, and impact of RIPK1 in CC cells and cell lines.
• RIPK1 expression was higher in tumor tissues than in paracancerous tissues.
• Poor prognosis of CC was linked to RIPK1 upregulation.
• RIPK1 enhanced CC cell migration, invasion, and growth by regulating NF-κB and TNF.

## Introduction

Cervical carcinoma (CC) is the fourth most common cancer type among women worldwide [1,2]. According to the International Agency for Research on Cancer, there were 604,100 new cases of cervical cancer in 2020 and 341,800 related deaths [3,4]. Surgery is indicated in the early stages, and although radiotherapy and chemotherapy are used in clinical practice, the response rate is limited in patients with advanced cervical cancer or those who develop metastasis. Therefore, further research on improved treatment approaches is necessary [5], [6], [7], [8]. The combination of targeted therapy and other therapies has emerged as a new model to treat advanced cervical cancer. Studies on the oncology, tumor biology, and tumor morphology of cervical cancer have opened new avenues for tumor diagnosis and therapy [9], [10], [11], [12]. However, the pathogenesis of CC remains unclear, and biomarkers for detecting various stages of cervical cancer remain unknown. Hence, identifying reliable immune molecular markers for the early detection of cancer lesions and cervical cancer is important [13], [14], [15].

Receptor-interacting protein kinase 1 (RIPK1) is a multi-domain protein that includes an N-terminal kinase domain, intermediate domain, and C-terminal death domain (DD) [16]. RIPK1 is a vital regulator of cell fate and functions as an administrator of RIPK3-mediated apoptosis as well as a mediator of caspase-8-mediated apoptosis [17], [18], [19]. Thu, RIPK1 is a viable therapeutic target for several neurological, cardiovascular, renal, and hepatic disorders, as well as inflammatory and infectious diseases [20]. In addition to its role in cell death, RIPK1 abrogates tumor necrosis factor (TNF) signaling via nuclear factor kappa-B (NF-κB) in cancer cells. This improves the ability of cancer cells to produce immunosuppressive chemokines and increases cancer cell survival. Cells lacking RIPK1 are highly sensitive to TNF-α-induced death, possibly due to the lack of RIPK1 and failure to activate the survival transcription factor NF-κB. The intermediate domain of RIPK1 is the structural basis for NF-κB activation [21]. RIPK1 involvement has been reported in breast cancer, esophageal squamous cell cancer, and lung cancer [22]. Furthermore, as RIPK1 influences cell death, it may be a true biomarker and novel target for cancer diagnosis and treatment [23], [24], [25].

NF-κB is a transcription factor involved in apoptosis, viral replication, tumorigenesis, inflammation, and various autoimmune diseases [26], and TNF is a proinflammatory cytokine that mediates cell survival or death [27]. The role of canonical NF-κB is complicated, and it has dual effects in cancer onset and development [28]. Canonical NF-κB promotes tumor cell proliferation, survival, vascular invasion, and tumor progression [29], [30]. By facilitating cell activity maintenance, stimulating invasiveness, and regulating metabolic changes, NF-κB integrates various environmental cues to aid tumor growth. Thus, NF-κB has attracted widespread attention in the treatment of tumor diseases.

## Materials and methods

### Patients and tissue samples

Clinical tissue samples from 50 patients with CC who had not received neoadjuvant anticancer therapy were collected between February 2019 and December 2020 at the First Affiliated Hospital of Bengbu Medical College Hospital, China. The ethics committee of the First Affiliated Hospital of Bengbu Medical College Hospital approved this study, which complied with the ethical principles of the Declaration of Helsinki. Written informed consent was obtained from all patients.

### Immunohistochemistry (IHC)

IHC for RIPK1 was performed using the streptavidin-peroxidase technique according to standard procedures. Fifty CC tissue samples and 20 paracancerous tissue samples were used for IHC. Antigen retrieval was performed using sodium citrate buffer (1 mM, pH 6.0). After sequential blocking with 3% hydrogen peroxide and 10% normal goat serum, the samples were incubated at 4 °C overnight with anti-RIPK1 antibody (ABclonal, Wuhan, China). After washing with phosphate-buffered saline (PBS), the samples were incubated with a secondary antibody and streptavidin-horseradish peroxidase (HRP) for 30 min and then reaction was detected using DAB. All results were independently interpreted by two pathologists. Five images (× 200) were randomly observed for each CC tissue sample, and 100 cells were selected for counting. The scores were calculated as the proportion of positive (PP) cells: PP below 25%, 25%–50%, 50%–75% and 75%–100% were assigned PP scores of 1, 2, 3, and 4, respectively. Yellow, yellow-brown, and dark-brown cell staining were defined as cell staining intensity (SI) scores of 1, 2, and 3, respectively. The protein expression intensity was quantitated using PP × SI. Samples were defined as having high RIPK1 expression when their score was > 6.

### Cell lines and culture

The Cell Bank of the Institute of Biochemistry and Cell Biology, China Academy of Sciences (Shanghai, China) provided two CC cell lines (SIHA and C33A) that were maintained in Minimum Essential Medium (MEM; Procell Life Science & Technology Co. Ltd., China) supplemented with 10% fetal bovine serum (FBS; Gibco, New Zealand), 100 U/ml penicillin, and 100 g/ml streptomycin at 37 °C in an incubator with 5% CO_2_.

### Cell transfection

Using Lipofectamine 2000 (Invitrogen, USA), RIPK1 siRNA was transfected with a non-specific sequence control (NC) generated by GeneChem (Shanghai, China). Cells were transfected according to the manufacturer's instructions with si-RIPK1#1: CCUUCAAGCCGGUCAAAUUTT; si-RIPK1#2: CUCAAGUACUGUAUCAGAATT; and si-RIPK1#3: CUACUAGACAGCACAAAUATT. The efficiency was verified using reverse transcription-quantitative polymerase chain reaction (RT-qPCR).

### RNA isolation and RT-qPCR

TRIzol was used to extract total RNA, which was then reverse-transcribed using the PrimeScript™ 1st Strand cDNA Synthesis Kit. SYBR Green Real-Time PCR Master Mix was used for conducting qPCR on a LightCycler 96 Real-Time PCR Detection System (Roche, USA). The qPCR program consisted of denaturing at 95 °C for 3 min followed by cycling at 95 °C for 5 min and then 60 °C for 30 s for 40 cycles. The following primers were used: *RIPK1*: F 5′-GGGAAGGTGTCTCTGTGTTTC-3′, R 5′-CCTCGTTGTGCTCAATGCAG-3′; and *GAPDH*: F 5′-CACCCACTCCTCCACCTTTG-3′, R 5′-CCACCACCCTGTTGCTGTAG-3′; *NF-κB*: F 5′-GGTGCGGCTCATGTTTACAG-3′, R 5′-GATGGCGTCTGATACCACGG-3′; *TNF-α*: F 5′-GAGGCCAAGCCCTGGTATG-3′, R 5′-CGGGCCGATTGATCTCAGC-3′. Primers were synthesized and purified by Jinkairui Biological Engineering Co., Ltd. (Wuhan, China). The 2^−ΔΔCt^ method was used to compare gene expression.

### Cell counting kit-8 (CCK-8) assay

The CCK-8 (Sigma-Aldrich, USA) assay was conducted to measure the effect of RIPK1 on CC cell proliferation. SIHA and C33A cells (3000 cells/well) transfected with si-RIPK1 and control cells were plated in 96‐well plates. After separate incubation for 24, 48, and 72 h, 10 μl CCK-8 assay solution was added to each well, and the plate was incubated at 37 °C for 1 h in the dark. An automatic microplate reader (BioTek Instruments Inc., Winooski, VT, USA) was used to measure the absorbance at 452 nm. This assay was conducted in triplicate.

### Colony formation assay

During the logarithmic growth period, cells (500 cells/well) were seeded in a 6-well plate. Staining was fixed in culture for approximately 2 weeks (when macroscopic cell colonies appeared). The cells were washed twice with PBS. The cells were fixed with a tissue fixative for 20 min, then stained with crystal violet dye (Beyotime, Shanghai, China) for 15 min and washed three times with PBS. Each well was photographed, counted, and analyzed using ImageJ.

### Wound-healing assay

Cells transfected with si-RIPK1 or control CC cells (2 × 10^5^ cells/well) were plated into 6-well plates. Next, a sterile plastic pipette tip was used to scratch the single-layer cells. The cells were subsequently cultured in serum-free media for 0, 24, or 48 h. Wound images were obtained using a light microscope (Olympus). The relative migration of each group was determined using ImageJ.

### Transwell invasion assay

Matrigel-coated Transwell (Corning, NY, USA, 8-μm pore) plates were used to seed cells in serum-free media. Additionally, 600 μl MEM supplemented with 10% FBS was added to the lower chamber. Cells were fixed in 4% formaldehyde, stained with 0.5% crystal violet, and then rinsed with PBS after 48 h of incubation. Three independent assays were subsequently conducted with five microscopic fields per hole, and ImageJ was used for analysis.

### Annexin V/propidium iodide (PI) apoptosis assay

Apoptosis assays were performed using a commercial kit (Procell Life Science & Technology Co., Ltd.). Briefly, 6-well plates were seeded with 5 × 10^5^ SIHA or C33A cells (control) and cells transfected with RIPK1 siRNA; cells were plated in triplicate. After 48 h, the cells were trypsinized and then suspended in PBS. The cells were then suspended in 500 µl binding buffer and incubated for 20 min in the dark with 5 µl annexin V FITC and 5 µl PI solutions. Flow cytometry (LSRFortessaTM X-20; BD Biosciences, San Jose, NJ, USA) was performed to evaluate the samples.

### Western blotting

The protein concentration was assessed using a BCA protein assay kit (Thermo Fisher Scientific, Grand Island, NY, USA).Normal and transfected cells were scraped on ice and collected via centrifugation (12,000 *g*, 10 min, 4 °C) for protein extraction. After mixing with the loading buffer, the protein sample was boiled for 8 min. The proteins were separated by performing sodium dodecyl sulfate-polyacrylamide gel electrophoresis using 10% resolving gels and the resolved protein bands were transferred onto polyvinylidene difluoride (PVDF) membranes. After blocking with 5% fat-free milk for 1 h at room temperature, the membranes were incubated with primary antibodies against RIPK1, NF-κB, TNF-α, and GAPDH (ABclonal, China) at 4 °C overnight. The membranes were then incubated with appropriate horseradish peroxidase-labeled secondary antibodies at room temperature for 2 h. Finally, the immunoreactive protein bands were quantified using ImageJ (Software Inquiry; Quebec, Canada) after being recorded using a Bio-Rad ChemiDoc XRS Imaging System (Bio-Rad Laboratories, CA, USA).

### Statistical analysis

All statistical analyses were conducted using GraphPad Prism 8.01 and SPSS 20.0. Data were analyzed using two-tailed paired and unpaired Student's *t*-tests, one-way analysis of variance, the Kaplan–Meier product-limit method, and the log-rank test. Data are presented as the mean ± standard deviation. P values are indicated as * *p* < 0.05, ** *p* < 0.01, and *** *p* < 0.001.

## Results

### RIPK1 is overexpressed in CC and associated with poor prognosis

**Fig. 1:**
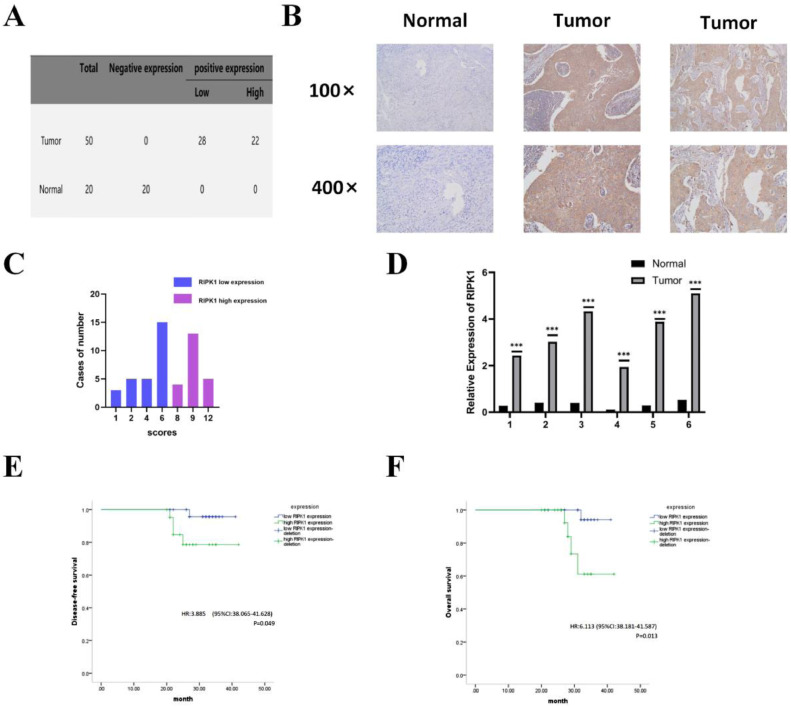
**In CC patients, RIPK1 is overexpressed and is linked to poor prognosis. A:** RIPK1 expression in 50 CC tissue samples and 20 paracancerous tissue samples. **B:** RIPK1 IHC staining in normal cervical and CC tissues. Magnifications: 100 × and 400 × . **C:** CC tissues stratified by the IHC staining index. **D:** Comparison of *RIPK1* mRNA expression in CC and paracancerous tissues using RT-qPCR. **E and F:** RIPK1 expression-based stratified Kaplan–Meier analysis of the DFS and OS in 50 CC patients.

RIPK1, receptor-interacting protein kinase 1; CC, cervical carcinoma; RT‐qPCR, reverse transcription-quantitative polymerase chain reaction; IHC, immunohistochemistry. *p < 0.05; **p < 0.01; ***p < 0.001.

First, we selected tumor (*n* = 50) and paracancerous (*n* = 20) tissues from patients with cervical cancer for IHC to clarify the function and clinical significance of RIPK1 in CC (Fig. 1A). Compared with that in paracancerous tissues, mean RIPK1 expression in CC tissues was significantly elevated (*P* < 0.001). RIPK1 was predominantly expressed in the cytoplasm (Fig. 1B), suggesting that cytoplasmic RIPK1 contributes toward CC development. According to the degree of immunostaining, the cells were divided into two groups: high expression (*n* = 22) and low expression (*n* = 28) (Fig. 1C). To determine whether RIPK1 was ectopically overexpressed in CC, we assessed the expression of *RIPK1* mRNA in six freshly frozen matched CC tissue samples. Similar to the IHC results, RIPK1 expression was upregulated in CC tissues than in paracancerous tissues (Fig. 1D). The effect of RIPK1 expression on disease-free survival (DFS) and overall survival (OS) in patients with CC was evaluated through a Kaplan–Meier survival analysis. Our results showed significantly shorter DFS (hazard ratio, 3.885; *p* = 0.049) (*p* < 0.05) and OS (hazard ratio, 6.113; *p* = 0.013) (*p* < 0.05) in patients with higher RIPK1 (Fig. 1E, F). Overall, these results showed that RIPK1 expression was elevated in CC tissues and was associated with poor prognosis in CC patients.

### *RIPK1* knockdown suppresses CC cell growth by inducing apoptosis

**Fig. 2:**
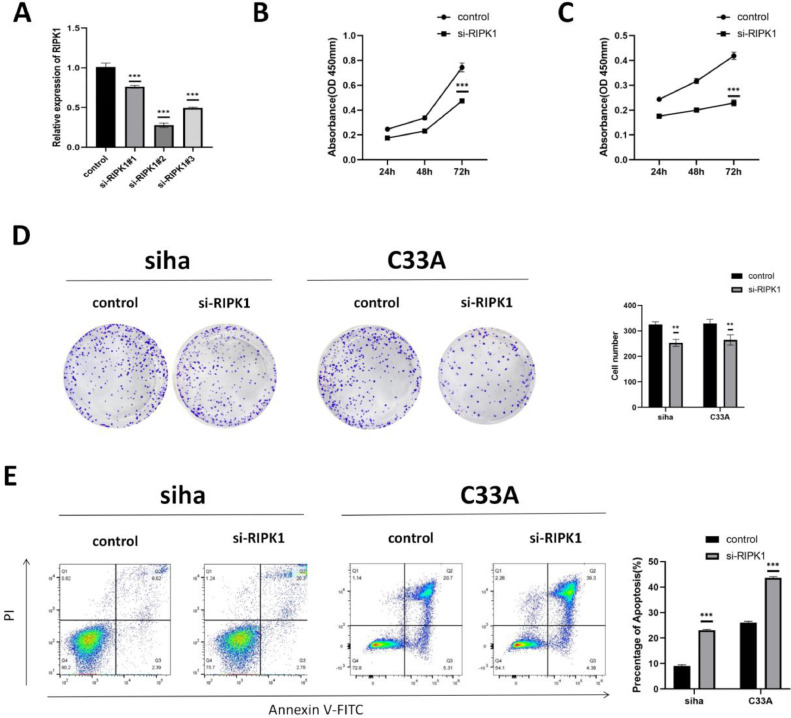
***RIPK1* knockdown increases apoptosis and suppresses CC cell proliferation. A:** RT-qPCR analyses of *RIPK1* expression in si-RIPK1-RNA-transfected SIHA and C33A cells. **B and C:** CCK-8 assay to determine the effects of *RIPK1* knockdown on SIHA and C33A cell proliferation. **D:** Clone formation assays to assess the effects of *RIPK1* knockdown on SIHA and C33A cell proliferation. **E:** Flow cytometry analysis of SIHA and C33A cell apoptosis with *RIPK1* knockdown. RIPK1, receptor-interacting protein kinase 1; RT‐qPCR, reverse transcription-quantitative polymerase chain reaction. *p < 0.05; **p < 0.01; ***p < 0.001.

Because of its increased expression, RIPK1 may play an important role in CC carcinogenesis. We knocked down *RIPK1* expression in SIHA and C33A cells to assess this hypothesis. We performed RT-qPCR to confirm downregulation of *RIPK1* expression in SIHA and C33A cells (*p* < 0.001), and si-RIPK1#2 was chosen for further experiments (Fig. 2A). CCK-8 assay showed that *RIPK1* knockdown significantly reduced the proliferation of SIHA and C33A cells (*p* < 0.001) (Fig. 2B and C). Colony formation assay revealed that *RIPK1* knockdown dramatically reduced the ability of SIHA and C33A cells to proliferate as single cells as well as their tumorigenicity, compared to that of control cells(Fig. 2D). Furthermore, we evaluated the effects of *RIPK1* knockdown on apoptosis using flow cytometry (Fig. 2E). When SIHA and C33A cells were transfected with si-RIPK1, apoptosis increased significantly compared to that of control cells. In conclusion, *RIPK1* knockdown prevented CC cell growth.

### *RIPK1* knockdown inhibits CC cell migration and invasion

**Fig. 3:**
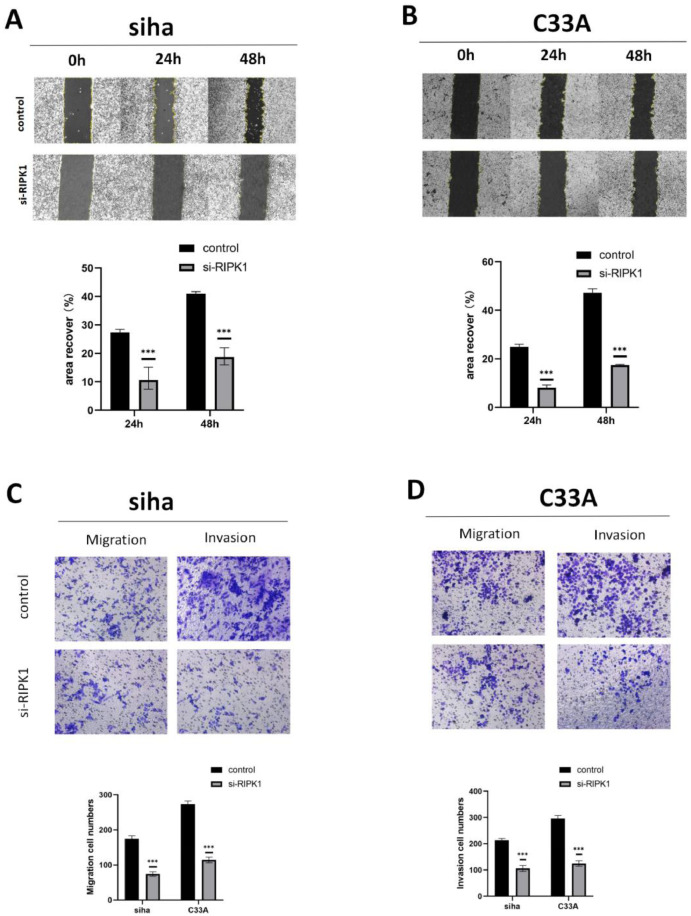
**CC cell invasion and migration are inhibited by RIPK1 knockdown in vitro. A:** The wound-healing assay revealed the effect of *RIPK1* knockdown on the migratory ability of si-RIPK1-transfected SIHA cells. **B:** The wound-healing assay revealed the effect of RIPK1 on the migratory ability of si-RIPK1-transfected C33A cells. **C:** Effect of *RIPK1* knockdown on SIHA cell invasion and migration evaluated using a Transwell assay. **D:** Effect of *RIPK1* knockdown on C33A cell invasion and migration evaluated using a Transwell assay. RIPK1, receptor-interacting protein kinase 1. *P < 0.05; **P < 0.01; ***P < 0.001.

The effect of RIPK1 on cell invasion and migration was investigated using Transwell migration and wound-healing assays, respectively. si-RIPK1-transfected SIHA and C33A cells demonstrated decreased migration rates than the control cells (*p* < 0.001) (Fig. 3A, B). The Transwell assay also revealed that *RIPK1* knockdown reduced the invasive capacity of SIHA and C33A cells (*p* < 0.001) (Fig. 3C, D). Overall, these results indicated that *RIPK1* knockdown inhibited the migration and invasion of CC cells.

### RIPK1 promotes CC cell proliferation, migration, invasion, and apoptosis potentially by regulating the NF-κB–TNF-α signaling pathway

**Fig. 4:**
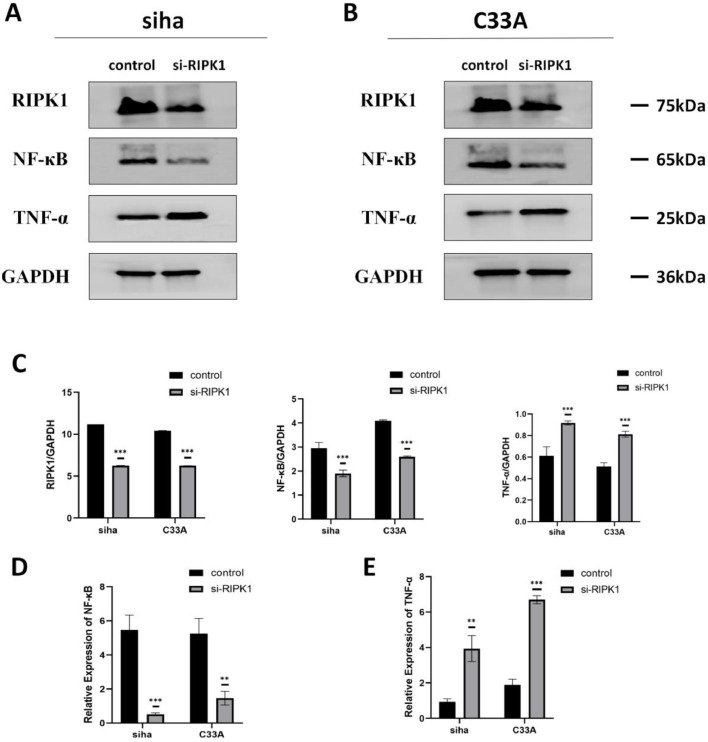
**RIPK1 can regulate the NF-κB–TNF**-α **signaling pathway. A, B, and C:** Western blot to assess NF-κB and TNF-α expression levels in SIHA and C33A cells transfected with si-RIPK1-RNA. **D and E:** RT-qPCR to verify NF-κB and TNF-α expression levels in SIHA and C33A cells transfected with si-RIPK1-RNA. RIPK1, receptor-interacting protein kinase 1. *P < 0.05; **P < 0.01; ***P < 0.001.

To elucidate whether RIPK1 stimulates CC cell growth by regulating NF-κB and TNF-α, we performed western blot analysis. We compared NF-κB and TNF-α levels between SIHA and C33A cells with *RIPK1* knockdown and control cells to confirm the mechanism underlying RIPK1 action in CC cells. *RIPK1* knockdown decreased NF-κB levels and increased TNF-α levels in CC cells (Fig. 4A-E). These findings demonstrated that RIPK1 potentially enhances CC cell proliferation, migration, and invasion by activating NF-κB and inhibiting TNF-α release.

## Discussion

In this study, we showed that RIPK1 promoted the growth of CC cells in vitro. Our results demonstrated the potential of RIPK1 as a novel therapeutic target and molecular marker for CC. RIPK1 expression was elevated in CC tissues and cells and was linked to poor DFS and OS. Additionally, RIPK1 prevented cell death while promoting CC growth and metastasis. Furthermore, the biological activities previously mentioned conflict with RIPK1 knockdown. Based on our findings we propose that RIPK1 plays an important role in CC and is linked to a poor prognosis, and thus, it may be a novel target and molecular diagnostic marker for CC therapy.

CC remains one of the most commonly diagnosed cancers [1,31]. Epidemiological studies have revealed that the age of CC onset is decreasing worldwide [32]. The survival rates of locally advanced disease and distant metastatic disease are 58% and 17%, respectively, and disease recurrence can be either local or distant [33]. Significant differences exist in local (10% stage IA, stage IVA II, 42%, 74%) and distant recurrences, which have been documented in 15–61% of patients at the initial stage of diagnosis [34]. However, recurrent and metastatic tumors remain refractory to treatment. Unchecked cancer cell growth and metastasis are the main causes of high mortality and recurrence rates; however, the molecular processes and pathways leading to CC carcinogenesis remain unknown.

By activating NF-κB, caspase-8, and reactive oxygen species generation, RIPK1 plays key roles in cell survival, apoptosis, and programmed necrosis [34]. The effectiveness of RIPK1 inhibitors in treating disorders has been demonstrated by inhibiting RIPK1 kinase activity using enhanced necrostatin-1 (R-7-Cl-O-Nec-1, Nec-1 s) and in animal models, such as RIPK1 kinase-dead mutant mice [35], [36], [37]. Proinflammatory cytokine production and cell death (apoptosis and necroptosis), which occur downstream of numerous signaling pathways are also regulated by RIPK1 [38]. RIPK1 is an upstream checkpoint for cell death and survival, serving as both a kinase and scaffold protein. Other immunological mechanisms such as NF-κB signaling and TNF-α-mediated classical apoptosis are promoted by its scaffold functions [39]. In cancer cells, TNF-α signaling is mediated via NF-κB and diverted from its role in cell death by RIPK1. This increases cancer cell survival by activating the immunosuppressive chemokine system. As RIPK1 prevents NF-κB activation, cells lacking RIPK1 are particularly vulnerable to TNF-α-induced cell death. The intermediate domain of RIPK1 is the structural basis of NF-κB activation [40]. Thus, further research is required to fully understand how RIPK1 affects CC development.

Here, we established that RIPK1 is overexpressed in CC tissues (Fig. 1A-D). IHC scores revealed that increased RIPK1 expression in CC was associated with poor DFS and OS (Fig. 1E, F). These findings support the notion that RIPK1 plays a crucial role in initiation and development of CC. RIPK1 knockdown reduced CC proliferation while increasing cell death in vitro. RIPK1 suppressed TNF-α release while activating NF-κB, which in turn increased CC proliferation, metastasis, and invasion while decreasing cell death. These findings demonstrate that RIPK1 significantly contributed to CC development and may therefore be a target for CC therapy.

In conclusion, our findings suggest that RIPK1 regulates NF-κB and TNF-α in vitro to promote CC cell proliferation, invasion, and migration, while inhibiting cell death. Therefore, *RIPK1* is a potential therapeutic target for CC while offering new insights into its prognosis. A more thorough understanding of RIPK1 function may help identify suitable treatment targets for CC.

## Funding

This study was supported by the 10.13039/501100007924Bengbu Medical College Natural Science Key Project (No. 2020byzd073).

## Author contribution statement

Conception and design: **WQB, SYG**. Development of methodology: **WQB, FJC, ZHW, XHG**. Analysis and interpretation of data: **WQB, FJC, ZHW, XJF, LZ**. Writing, review, and/or revision of the manuscript: **WQB, FJC, ZHW**. Study supervision: **WQB, LZ, SYG**. All authors accept the responsibility for the content presented in this manuscript and approve its submission.

## Ethics approval and consent to participate

The authors are accountable for all aspects of the work in ensuring that questions related to the accuracy or integrity of any part of the work are appropriately investigated and resolved. The study was conducted in accordance with the Declaration of Helsinki (as revised in 2013). The study was approved by the Ethics Committee of The First Affiliated Hospital of Bengbu Medical College (No. 143,2021), and all the patients signed the informed consent form.

## Availability of data and materials

All relevant data and materials have been published in the article. Further inquiries can be directed to the corresponding authors.

## Declaration of Competing Interest

The authors declare that they have no known competing financial interests or personal relationships that could have appeared to influence the work reported in this paper.
